# Dysregulated B cell differentiation towards antibody-secreting cells in neuromyelitis optica spectrum disorder

**DOI:** 10.1186/s12974-021-02375-w

**Published:** 2022-01-06

**Authors:** Yasunobu Hoshino, Daisuke Noto, Shuhei Sano, Yuji Tomizawa, Kazumasa Yokoyama, Nobutaka Hattori, Sachiko Miyake

**Affiliations:** 1 Department of Immunology, Juntendo University School of Medicine, 2-1-1 Hongo, Bunkyo-ku, Tokyo, 113-8421 Japan; 2 Department of Neurology, Juntendo University School of Medicine, Tokyo, Japan

**Keywords:** Neuromyelitis optica spectrum disorder, Autoantibody, B cells, IL-2

## Abstract

**Background:**

Anti-aquaporin 4 (AQP4) antibody (AQP4-Ab) is involved in the pathogenesis of neuromyelitis optica spectrum disorder (NMOSD). However, the mechanism involved in AQP4-Ab production remains unclear.

**Methods:**

We analyzed the immunophenotypes of patients with NMOSD and other neuroinflammatory diseases as well as healthy controls (HC) using flow cytometry. Transcriptome analysis of B cell subsets obtained from NMOSD patients and HCs was performed. The differentiation capacity of B cell subsets into antibody-secreting cells was analyzed.

**Results:**

The frequencies of switched memory B (SMB) cells and plasmablasts were increased and that of naïve B cells was decreased in NMOSD patients compared with relapsing–remitting multiple sclerosis patients and HC. SMB cells from NMOSD patients had an enhanced potential to differentiate into antibody-secreting cells when cocultured with T peripheral helper cells. Transcriptome analysis revealed that the profiles of B cell lineage transcription factors in NMOSD were skewed towards antibody-secreting cells and that IL-2 signaling was upregulated, particularly in naïve B cells. Naïve B cells expressing CD25, a receptor of IL-2, were increased in NMOSD patients and had a higher potential to differentiate into antibody-secreting cells, suggesting CD25^+^ naïve B cells are committed to differentiate into antibody-secreting cells.

**Conclusions:**

To the best of our knowledge, this is the first study to demonstrate that B cells in NMOSD patients are abnormally skewed towards antibody-secreting cells at the transcriptome level during the early differentiation phase, and that IL-2 might participate in this pathogenic process. Our study indicates that CD25^+^ naïve B cells are a novel candidate precursor of antibody-secreting cells in autoimmune diseases.

**Supplementary Information:**

The online version contains supplementary material available at 10.1186/s12974-021-02375-w.

## Background

Neuromyelitis optica spectrum disorder (NMOSD) is an autoimmune disease of the central nervous system (CNS) characterized by severe optic neuritis, myelitis, and the presence of anti-aquaporin 4 (AQP4) antibody (AQP4-Ab) [1, 2]. The pathogenicity of AQP4-Ab was further shown by several studies using rodents [3–5] and supported by clinical findings including the therapeutic efficacy of plasma exchange, complement inhibitors, and B cell-depleting therapy [6–8]. However, the mechanisms involved in autoantibody production remain unclear. Previous studies showed that the frequencies of plasmablasts in the peripheral blood and cerebrospinal fluid (CSF) of NMOSD patients were increased [9, 10]. Recent studies indicated that naïve B cells from NMOSD patients produced autoantibodies or AQP4-Abs, suggesting a deficiency of central tolerance in NMOSD patients [11, 12].

The production of autoantibodies and defects in B cell tolerance are associated with the pathomechanisms of several autoimmune diseases. Many studies have reported that autoreactive naïve B cells are increased in various autoimmune diseases including NMOSD, suggesting defects in early B cell tolerance checkpoints [13, 14]. Recently, CD27^−^IgD^−^CXCR5^−^CD11c^+^ B cells (DN2 cells) expanded in patients with systemic lupus erythematosus (SLE) were shown to differentiate into antibody-secreting cells during the extrafollicular response [15]. DN2 cells were reported to be derived from CD27^−^IgD^+^CXCR5^−^CD11c^+^ cells, termed activated naïve B cells. These studies suggested that abnormalities of early B cell development may exist in autoimmune pathology.

In this study, we found a decrease in the number of naïve B cells and an increase in switched memory B (SMB) cells and plasmablasts in NMOSD patients compared with healthy controls (HC). Transcriptome analysis of B cell subsets in NMOSD patients revealed that the profiles of B cell lineage transcription factors were skewed towards an antibody-secreting cell-like phenotype. In accordance with this finding, SMB cells from NMOSD patients had a higher potential to differentiate into antibody-secreting cells when cocultured with T cells compared with those from HC. Furthermore, transcriptome analysis revealed that IL-2 signaling was activated, particularly in naïve B cells from NMOSD patients. Indeed, numbers of naïve B cells expressing CD25, a receptor of IL-2, were increased in NMOSD patients and CD25^+^ naïve B cells exhibited a higher potential to differentiate into antibody-secreting cells compared with CD25^−^ naïve B cells, suggesting that CD25^+^ naïve B cells are committed to differentiate into antibody-secreting cells. Our results indicated that CD25^+^ naïve B cells are a novel candidate precursor antibody-secreting cell.

## Methods

### Patients and controls

Blood was obtained from 24 patients with AQP4-Ab positive NMOSD, 22 patients with multiple sclerosis (MS), 11 patients with myelin oligodendrocyte glycoprotein antibody-associated disease (MOG-AD) and 27 HC. CSF was obtained from 8 patients with NMOSD, 9 patients with MS, and 12 non-inflammatory disease controls (DC) (Table 1). All NMOSD patients met the 2015 NMOSD diagnostic criteria [16]. All patients with MS had relapsing–remitting MS and had clinically definite MS according to the 2017 McDonald MS Diagnostic Criteria [17]. Nine patients with MOG-AD fulfilled the 2015 NMOSD diagnostic criteria. The other three did not meet the NMOSD criteria, because they did not develop myelitis *and* optic neuritis (optic neuritis alone *n* = 2; myelitis alone *n* = 1). Serum MOG-Ab levels were measured using a cell-based assay at Tohoku University (Sendai, Japan). For the flow cytometric analysis of CD25^+^ or CD25^−^ populations in B cell subsets, we recruited nine female patients with NMOSD (median age 48.0 years, interquartile range 38.0–54.5) and nine age- and sex-matched HC (median age 47.0 years, interquartile range 39.0–52.5).

**Table 1:** Characteristics HCs, DCs, and patients with MS, NMOSD, and MOG-AD

	HC	MS	NMOSD	MOG-AD	DC (CSF)	MS (CSF)	NMOSD (CSF)
Number	27	22	24	11	12	9	8
Age	38 (22–60)	34 (22–54)	46 (24–65)	36 (17–66)	51 (30–72)	39 (23–53)	43 (39–74)
Male: female	6:21	5:17	2:22	3:8	6:6	2:7	1:7
disease duration (months)		56 (0–118)	53 (0–173)	35 (0–78)		51 (0–118)	47 (0–162)
Age of symptom onset		29 (19–52)	38 (20–63)	50 (16–66)		33 (19–52)	39 (26–63)
Medications							
Naïve		15	0	4		6	1
IFNβ, *n*		4				2	
Glatiramer acetate, *n*		2				0	
Dymethyl fumarate, *n*		1				1	
Prednisone, *n*		0	22	7		0	5
Daily dose of prednisone, median (mg/day)			7	5 (3–7)			8 (5–14)
Tacrolimus, *n*		0	4	0		0	3
Azathioprine, *n*		0	4	0		0	0
Mizoribine, *n*		0	1	0		0	0
					Normal pressure hydrocephalus *n* = 1 Cerebral venous thrombosis *n* = 1 Old cerebral infarction *n* = 4 causeless sensory abnormality *n* = 2 Parkinson disease *n* = 4		

*IFNβ*: interferon-β

### Flow cytometry and cell sorting

Peripheral blood mononuclear cells (PBMCs) were isolated from whole blood by density centrifugation using Histopaque-1077 (Merck, Darmstadt, Germany). PBMCs were stained with antibodies listed in Additional file 4 Table S1. Data were acquired on a BD LSRFortessa (BD Bioscience, Franklin Lakes, NJ, USA) and analyzed using FlowJo 10.6.1 (FlowJo LLC, Ashland, OR, USA).

### Cell culture

B cells were isolated from PBMCs by anti-CD19 magnetic bead (Miltenyi Biotec, Bergisch Gladbach, Germany) positive selection. The cells were sorted on a BD FACSAria Fusion (BD bioscience). Sorted B cell populations were cocultured with autologous T cell populations at a ratio of 5:1 in 200 μl of RPMI-1640 supplemented with 10% fetal bovine serum, 2 mM l-glutamine, 50 U/ml penicillin, and 50 mg/ml streptomycin (all from Thermo Fisher, Waltham, MA, USA), and stimulated with lipopolysaccharide (5 μg/ml, Chondrex, Woodinville, WA, USA) and staphylococcal enterotoxin B (1 μg/ml, Sigma-Aldrich, St. Louis, MO, USA) for 7 days. For blocking experiments, IL-21R-Fc Chimera Protein (10 μg/ml, R&D Systems, Minneapolis, MN, USA) and Human IgG1-Fc Protein (10 μg/ml, R&D Systems) as a control, were used. For B cell cultures, sorted cells were stimulated with F(ab’)_2_ fragment goat anti-human IgG + IgM (H + L) (Jackson ImmunoResearch, West Grove, PA, USA), MEGACD40L (Enzo Life Sciences, Farmingdale, NY, USA), and recombinant human IL-21 (BioLegend, San Diego, CA, USA) for 7 days.

### Measurement of IgG

IgG secreted into the culture supernatant was quantitated by sandwich ELISA using an IgG (Total) Human ELISA Kit (Thermo Fisher) according to the manufacturer’s instructions.

### RNA-seq analysis

Total RNA was isolated and purified from sorted cells using an RNeasy Micro Kit (Qiagen, Hilden, Germany). RNA-seq libraries were generated with the Ovation SoLo RNA-Seq System, Human kit (NuGEN, Redwood City, CA, USA) using 5 ng of total RNA. The cDNA libraries were sequenced by 50-base single-read sequencing on an Illumina HiSeq 2500 sequencer (Illumina, San Diego, CA, USA). The sequencing run and the base call analysis were performed according to the HiSeq 2500 System Guide with TruSeq SBS kit v3-HS. Raw sequence data were generated with processing by CASAVA-1.8.4 with version RTA 1.17.20.0. Reads were mapped to the hg38 genome with Tophat2. Normalized FPKM values and differential gene expression analyses were generated with Cuffdiff2. *Q* values (the FDR-adjusted *p* value after Benjamini–Hochberg correction for multi-testing) lower than 0.05 were considered significant.

### RT-qPCR

cDNA was synthesized from 500 ng total RNA using ReverTra Ace qPCR RT Master Mix (Toyobo, Osaka, Japan). Real-time quantitative PCR (RT-qPCR) was performed using a 7500 Fast Real-Time PCR System (Applied Biosystems, Foster City, CA, USA) with Fast SYBR Green Master Mix (Thermo Fisher Scientific). mRNA levels were normalized to beta-actin (ACTB) in each sample. The specific primers used in this study are listed in Additional file 4: Table S2.

### Statistical analysis

Data were analyzed using Prism 7 software (GraphPad Software, La Jolla, CA, USA) and differences between groups were analyzed using the Kruskal–Wallis test followed by Dunn’s multiple comparisons test, the Mann–Whitney *U* test, or the Wilcoxon matched-pairs test. The significance level was set at *P* < 0.05. Correlations between two variables were analyzed using Spearman’s rank correlation test.

## Results

### Alteration of B cell subsets in the peripheral blood and cerebrospinal fluid of NMOSD patients

**Fig. 1:**
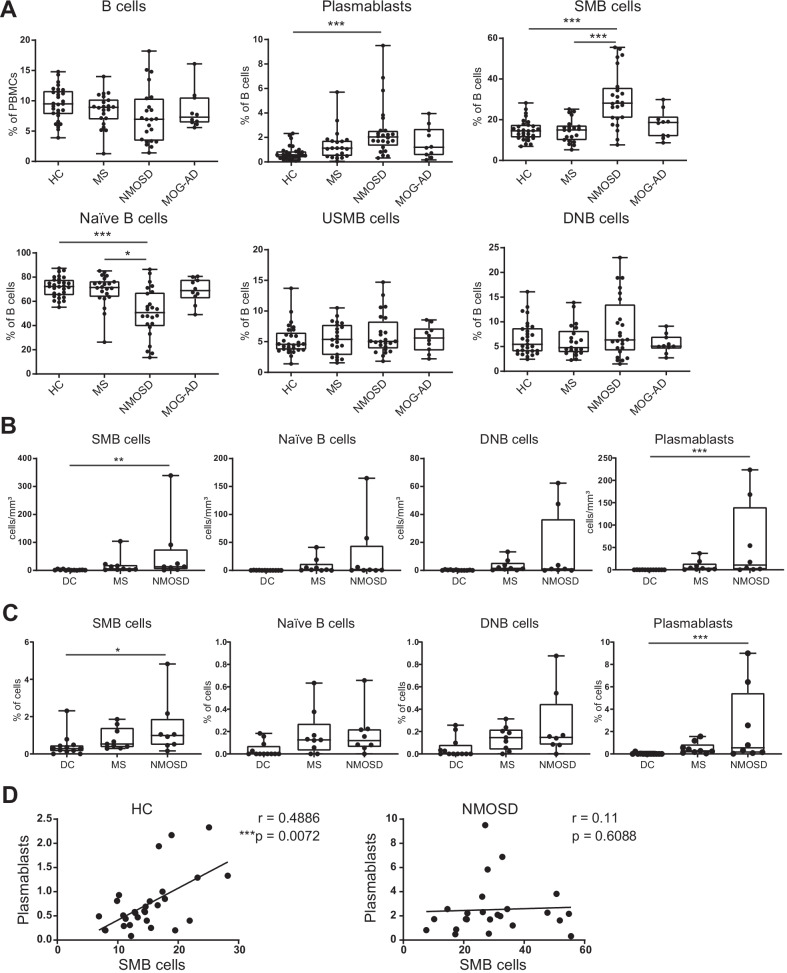
Increased numbers of SMB cells and plasmablasts in the peripheral blood of AQP4-Ab positive NMOSD patients. **A** Frequencies of B cell subsets among PBMCs or CD19^+^ B cells (Kruskal–Wallis test). **B** Comparisons of the numbers of cells in the CSF of DC, MS, and AQP-4-Ab positive NMOSD patients (Kruskal–Wallis test). **C** Comparisons of the proportion of cells in the CSF of DC, MS, and AQP-4-Ab positive NMOSD patients (Kruskal–Wallis test). **D** Correlations between the frequencies of SMB cells and plasmablasts in each group (Spearman’s correlation). The box plot indicates the first and third quartiles and the middle line indicates the median. Whiskers indicate the minimum and maximum. *P < 0.05, **P < 0.01, ***P < 0.005

We analyzed B cell populations in peripheral blood obtained from patients with autoimmune neurological disease without relapse for at least 6 months (Table 1). Plasmablasts (CD19^+^CD20^−^CD27^hi^CD38^hi^CD180^−^) and SMB cells (CD19^+^CD20^+^CD27^+^IgD^−^) were significantly increased in NMOSD compared with HC, whereas there was no difference in the proportion of total B cells in PBMCs among HCs and disease groups (Fig. 1A; Additional file 1: Fig. S1A). In contrast, naïve B cells (CD19^+^CD20^+^CD27^−^IgD^+^) were decreased in NMOSD compared with HC and MS. A limitation of this study was excluding the effects of corticosteroid therapy on patients with NMOSD. However, changes in the frequencies of B cell subsets in NMOSD were not observed in patients with MOG-AD, most of whom were treated with corticosteroids, suggesting that differences in the B cell subsets in NMOSD could not be explained by the effect of the corticosteroid therapy. Recently, double-negative B cells (CD19^+^CD20^+^CD27^−^IgD^−^, DNB cells) that were increased in SLE patients were reported to differentiate into antibody-secreting cells [15]. In our study, there were no statistically significant changes between the HC and disease groups. We found similar tendencies in the frequencies of each B cell subset in PBMCs (Additional file 1: Fig. S1B). Thus, we assumed that the increase of SMB cells and plasmablasts and the decrease of naïve B cells were characteristic features of NMOSD. Next, we investigated the CSF from patients with NMOSD or MS during disease relapse as well as from patients without inflammatory diseases as a DC (Table 1). In accordance with the peripheral blood analysis, the numbers and proportions of plasmablasts and SMB cells were significantly increased in the CSF of patients with NMOSD compared with DC (Fig. 1B, C). To reveal an association between plasmablasts and SMB cells, we analyzed correlations between the frequencies of SMB cells and plasmablasts in each group. As expected, we found that the frequencies of SMB and plasmablasts were strongly correlated in HC (Fig. 1D). However, this positive correlation between the frequencies of SMB cells and plasmablasts was not observed in patients with NMOSD, suggesting that the differentiation of B cells may be altered.

### Transcriptional profiles of B cell subsets are dysregulated in NMOSD

**Fig. 2:**
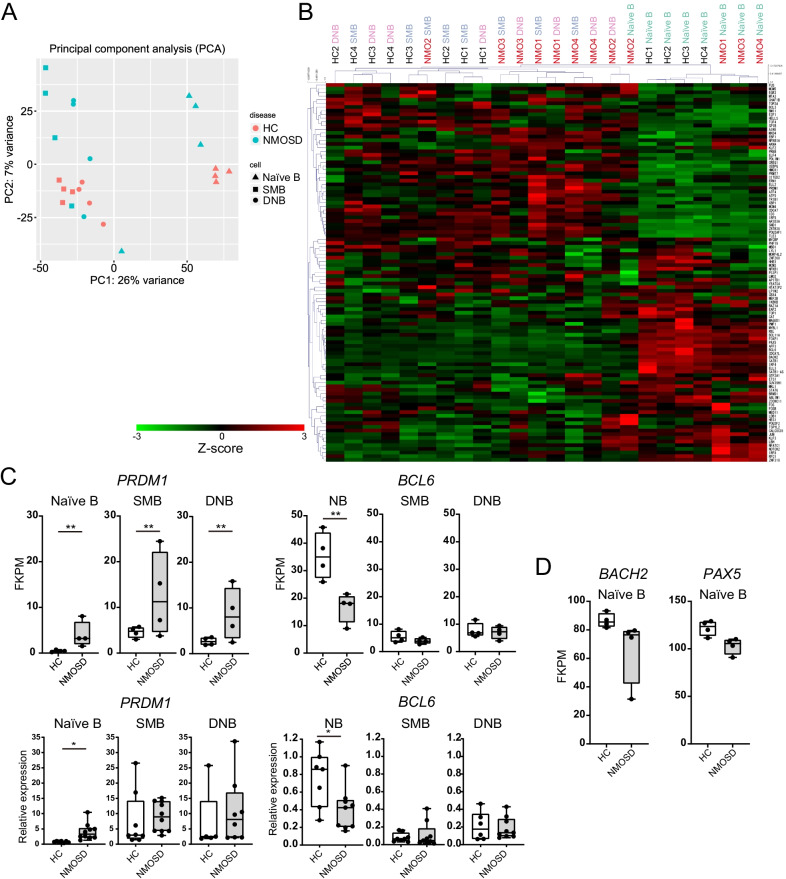
Dysregulated transcriptional profiles of B cell subsets in AQP4-Ab positive NMOSD patients. **A** Principal component analysis of transcriptional profiles in B cell subsets obtained from HCs and AQP4-Ab positive NMOSD patients. **B** Expression profiles of differentially-expressed transcription factors in B cell subsets. Relative expression levels (Z-scores) of genes are shown in a heatmap. **C** (upper row) Expression levels (FKPM: fragments per kilobase of transcript per million) of PRDM1 and BCL6 in each B cell subset (**Q < 0.01, FDR-adjusted p-value after Benjamini–Hochberg correction for multi-testing). (lower row) Relative expression analyzed by qPCR of PRDM1 and BCL6 in each B cell subset. **D** Expression levels (FKPM) of BACH2 and PAX5 in naïve B cells. **C**‒**D** The box plot indicates the first and third quartiles and the middle line indicates the median. Whiskers indicate the minimum and maximum

The alterations of B cell subsets led us to perform transcriptome profiling of naïve B, SMB, and DNB cells obtained from four NMOSD patients and four HCs using RNA-seq analysis. We compared the transcriptomic profiles of each B cell subset between NMOSD and HC, and detected 533 differentially expressed genes (DEGs) in naïve B cells, 218 DEGs in SMB cells, and 265 DEGs in DNB cells. Principal component analysis (PCA) demonstrated that each subset of B cells from NMOSD and HC was plotted separately, suggesting that the transcriptomic profiles in each B cell subset were different between NMOSD patients and HCs (Fig. 2A). To reveal the differentiation status of each B cell subset, we analyzed the expression levels of transcription factor genes that were expressed specifically in each B cell subset (Fig. 2B) [18]. Hierarchical clustering analysis showed that the expression patterns of these B cell transcription factors were divided into two major groups, naïve B cells and SMB-DNB. In each major group, the expression patterns of HC and NMOSD were separately clustered indicating a difference in the expression patterns between HC and NMOSD. In the SMB-DNB group, the difference between SMB cells and DNB cells tended to be smaller than the interindividual differences in each subset, suggesting SMB cells and DNB cells exist at a similar differentiation stage. We also found that the key transcription factor of plasmablasts, PRDM1 (also known as BLIMP-1), was upregulated in all three B cell subsets of NMOSD patients compared with those of HCs (Fig. 2B, C). Reciprocally, in NMOSD, the expression level of BCL6, an important transcription factor that maintains B cell fate, was downregulated, especially in naïve B cells. We confirmed the differential expressions of these transcription factors among B cell subsets using quantitative PCR (qPCR) analysis (Fig. 2C). Furthermore, the expressions of BACH2 and PAX5, important transcription factors for B cell development that repress the expression of PRDM1, tended to be decreased in naïve B cells from NMOSD patients (Fig. 2D).

### Enhanced differentiation of SMB cells into antibody-secreting cells

**Fig. 3:**
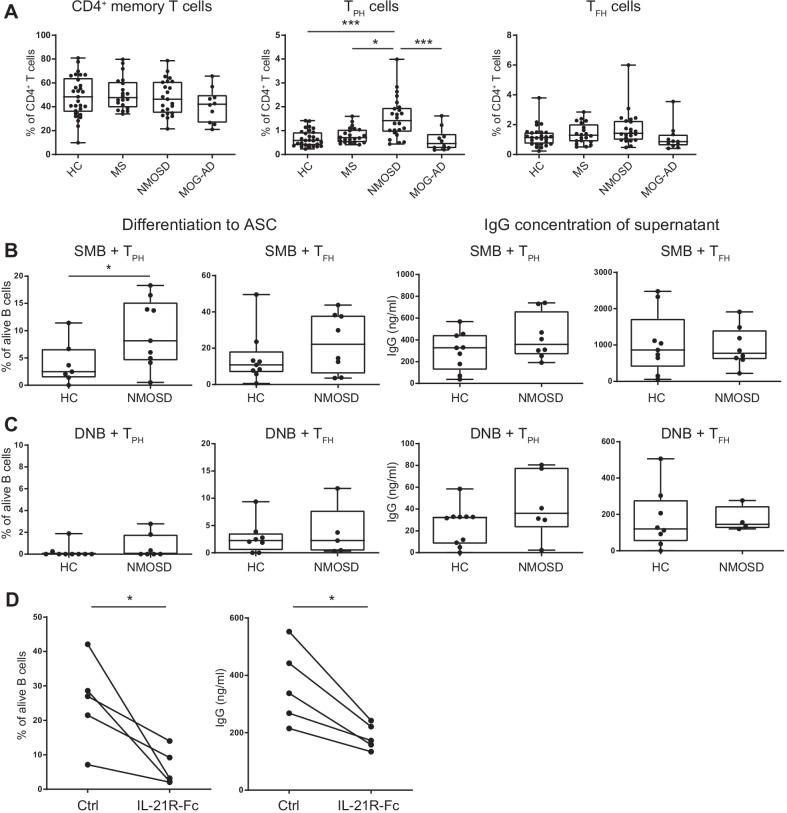
Significantly increased SMB cell differentiation to antibody-secreting cells by coculture with AQP4-Ab positive NMOSD T_PH_ cells. **A** Frequencies of T cell subsets among CD4^+^ T cells in HCs and patients (*P < 0.05, ***P < 0.005 by Kruskal–Wallis test). **B** Proportion of antibody-secreting cells differentiated from SMB cells cocultured with T_PH_ cells or T_FH_ cells and concentrations of IgG in supernatants. SMB cells, T_PH_ cells, and T_FH_ cells were sorted from the peripheral blood of HC or NMOSD patients, and each B cell subset was cocultured with T_PH_ cells or T_FH_ cells for 7 days. Then, the proportion of antibody-secreting cells was analyzed by flow cytometry and the concentration of IgG in culture supernatant was measured by ELISA (*P < 0.05, Mann–Whitney *U*-test). **C** Proportion of antibody-secreting cells differentiated from DNB cells cocultured with T_PH_ cells or T_FH_ cells and concentrations of IgG in supernatants. **D** Proportion of antibody-secreting cells in SMB cell and T_PH_ cell coculture with IL-21R-Fc or control IgG-Fc and concentration of IgG in supernatants from SMB cells and T_PH_ cell coculture with IL-21R-Fc or control IgG-Fc (*P < 0.05, Wilcoxon matched pairs test). The box plot indicates the first and third quartiles and the middle line indicates the median. Whiskers indicate the minimum and maximum

The increased expression of PRDM1 suggested the enhanced differentiation of antibody-secreting cells, including plasmablasts and plasma cells. Therefore, we investigated the ability of SMB cells and DNB cells to differentiate into antibody-secreting cells (CD19^+^CD20^lo^CD27^hi^CD38^hi^). In germinal centers, B cells are thought to be activated by T follicular helper (CD3^+^CD4^+^CD45RA^−^CXCR5^+^PD-1^hi^, T_FH_) cells through CD40–CD40L interactions and IL-21 secretion to produce more effective antibodies with a higher affinity. T_FH_ cells were reported to be increased in the peripheral blood of several autoimmune diseases including during the relapse phase of NMOSD patients [19, 20]. Recently, T peripheral helper (CD3^+^CD4^+^CD45RA^−^CXCR5^−^PD-1^hi^, T_PH_) cells were identified as helper T cells that promote antibody production from B cells in the inflamed tissues of patients with rheumatoid arthritis and other autoimmune diseases [21–24]. The frequency of T_PH_ cells was significantly increased in patients with NMOSD compared with HC as well as MS and MOG-AD patients, whereas the frequency of memory CD4^+^ T cells and T_FH_ cells was similar between the HC and disease groups (Fig. 3A; Additional file 2: Fig. S2A). We also found similar tendencies in the frequencies of each T cell subset in PBMCs (Additional file 2: Fig. S2B). We assumed that T_PH_ cells and T_FH_ cells may contribute to the differentiation of antibody-secreting cells in NMOSD and investigated the differentiation and immunoglobulin production of B cells cocultured with autologous T_PH_ cells or T_FH_ cells. When cocultured with T_PH_ cells, the proportion of antibody-secreting cells from SMB cells was significantly increased and the concentration of IgG in the coculture supernatant tended to be increased in the NMOSD group compared with the HC group (Fig. 3B). When SMB cells were cocultured with T_FH_ cells, the proportion of antibody-secreting cells tended to be increased in the NMOSD group compared with the HC group, but the concentration of IgG in the coculture supernatant was not different between both groups. DNB cells cocultured with T_PH_ cells and T_FH_ cells showed slight differentiation into antibody-secreting cells; however, the frequencies of these populations were lower than SMB cells and there was no difference between HC and NMOSD groups (Fig. 3C). These results suggested that SMB cells and DNB cells can differentiate into antibody-secreting cells when cocultured with T_PH_ cells or T_FH_ cells, and that SMB cells from NMOSD patients have a greater differentiation capacity than those from HCs. To assess the mechanism involved in SMB cell differentiation, we blocked IL-21 in the coculture of SMB cells and T_PH_ cells with an IL-21R-Fc chimera protein. The differentiation of SMB cells into antibody-secreting cells and IgG production were suppressed by the addition of IL-21R-Fc chimera protein, indicating that IL-21 might have an important role in the differentiation of SMB cells into antibody-secreting cells with help from T_PH_ cells (Fig. 3D).

### IL-2 signaling is activated in naïve B cells from NMOSD patients

**Fig. 4:**
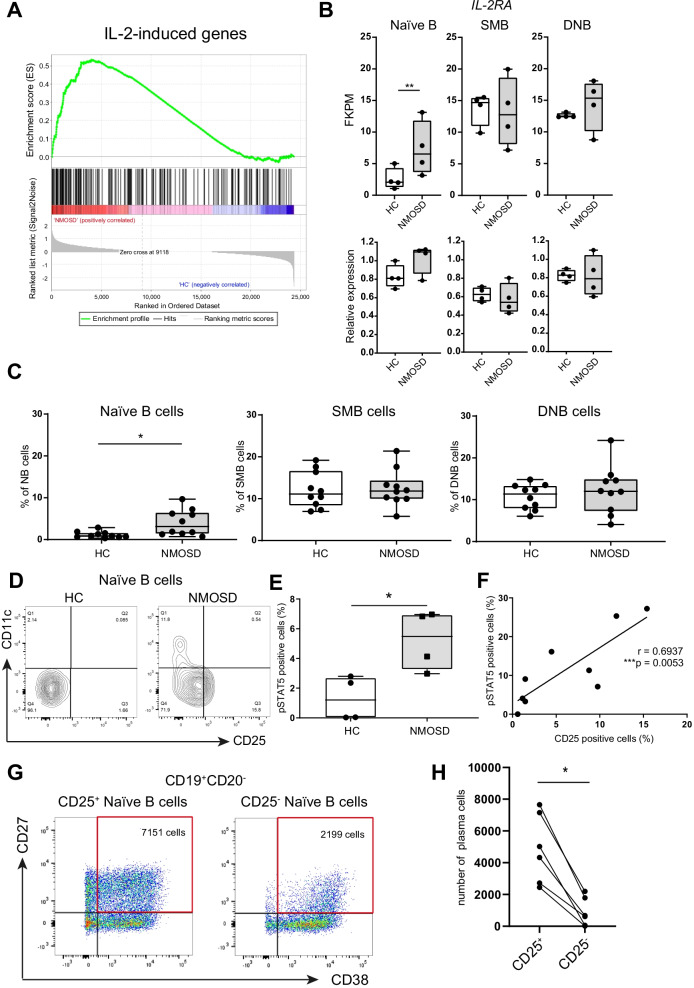
CD25^+^ naïve B cells in NMOSD show higher potential to differentiate into antibody-secreting cells. **A** Results of Gene Set Enrichment Analysis (GSEA) for IL-2-induced genes. **B** Expression level (FKPM) and relative expression of IL-2RA in each B cell subset. **C** Frequencies of CD25^+^ cells in each B cell subset (* *P* < 0.05, Mann–Whitney *U*-test). **D** CD25 and CD11b expressions in naïve B cells from HC and NMOSD analyzed using flow cytometry. **E** Frequency of pSTAT5 positive cells in naïve B cells (* *P* < 0.05, Mann–Whitney *U*-test). **F** Correlations between frequencies of CD25^+^ cells and pSTAT5 positive cells in naïve B cells of NMOSD (Spearman’s correlation). **G** Sorted CD25^+^ or CD25^−^ naïve B cells were cultured with a BCR cross-linking antibody, CD40L, IL-21, and IL-2 for 1 week. **H** Number of antibody-secreting cells in CD25^+^ or CD25^−^ naïve B cell culture (*P < 0.05, Wilcoxon matched pairs test)

Our transcriptome analysis revealed that B cells from NMOSD patients were skewed toward an antibody-secreting cell phenotype. To assess the mechanism involved in the transcriptional dysregulation of B cells in NMOSD patients, we performed Gene Set Enrichment Analysis. Surprisingly, we found that IL-2-induced genes were upregulated in the naïve B cells of NMOSD patients compared with those of HCs (Fig. 4A). Recently, transient IL-2 signals were reported to induce human naïve B cells to commit to plasma cell differentiation in vitro [25]. We evaluated the expression level of IL2RA in each B cell subset from NMOSD patients and found that its expression was significantly upregulated in naïve B cells of NMOSD patients compared with those of HCs (Fig. 4B). Next, we analyzed the expression of CD25 in B cell subsets using flow cytometry, and revealed that the CD25^+^ population was increased in naïve B cells from NMOSD patients (Fig. 4C). When we analyzed the frequencies of each CD25^+^ population in PBMCs, the frequency of the CD25^+^ population tended to increase in each population (Additional file 3: Fig. S3). Recently, CD27^−^IgD^+^CXCR5^−^CD11c^+^ activated naïve B cells, which are thought to be precursors of autoreactive DN2 cells, were reported to be increased in patients with SLE [15]. Our flow cytometry analysis confirmed that CD25^+^ naïve B cells in NMOSD patients were distinct from the population of CD11c^+^ activated naïve B cells (Fig. 4D). To investigate the activity of the IL-2 signaling pathway in naïve B cells, the frequency of phosphorylated signal transducer and activator of transcription 5 (pSTAT5) positive cells among naïve B cells was evaluated (Fig. 4E). We revealed that the frequency of pSTAT5 positive cells among naïve B cells in NMOSD patients was significantly higher than those in HC. Furthermore, the frequency of pSTAT5 positive cells in NMOSD was positively correlated with the frequency of CD25^+^ cells (Fig. 4F).

### CD25^+^ naïve B cells from NMOSD patients differentiate into antibody-secreting cells

We assumed that CD25^+^ naïve B cells from NMOSD patients were committed to differentiating into antibody-secreting cells. To confirm this hypothesis, CD25^+^ or CD25^−^ naïve B cells from NMOSD patients were sorted and cultured with B cell receptor (BCR) stimulation by BCR cross-linking antibody, CD40L, IL-21, and IL-2. After 1 week of culture, we found that the number of antibody-secreting cells from CD25^+^ naïve B cells was significantly higher than that from CD25^−^ naïve B cells (Fig. 4G, H). This result indicated that CD25^+^ naïve B cells were committed to differentiating into antibody-secreting cells.

## Discussion

In this study, we showed that the expressions of the key transcription factors of B cell lineage, BCL6, PAX5, and BACH2 were reduced and the expression of PRDM1, an essential transcription factor for differentiation into antibody-secreting cells, was significantly upregulated in NMOSD patients. BCL6 was reported to repress the expression of PRDM1 to maintain B cell fate [26, 27]. PAX5 is an important transcription factor that maintains B cell identity and induces the expression of transcription factors, such as BACH2, which is associated with maintaining B cell fate [28, 29]. BACH2 inhibits differentiation into antibody-secreting cells by repressing the expression of PRDM1 [30]. Our results suggested that the regulation of transcription factors that control B cell fate was altered, and that the transcriptional profiles of non-memory B cells in NMOSD patients were already skewed towards antibody-secreting cells. Recently, it was reported that naïve B cells from NMOSD patients differentiated into antibody-secreting cells, which secreted AQP4-Ab in vitro [11]. Another study showed that naïve B cells from NMOSD patients contained significantly higher frequencies of autoreactive B cells compared with HCs, suggesting impaired B cell tolerance in the early phase of B cell maturation [12]. In accord with these studies, the abnormality of transcriptional factors in naïve B cells suggested the expansion of an autoreactive subpopulation in NMOSD naïve B cells, which are committed to differentiate into antibody-secreting cells.

IL-2 regulates the differentiation and function of CD4^+^ T cells, especially regulatory T cells [31]. However, the roles of IL-2 in B cell-differentiation and function, particularly in autoimmune conditions, are unknown. IL-2-stimulation following stimulation with BCR cross-linking by CpG and CD40L repressed the expression of BACH2 and primed naïve B cells obtained from HCs to differentiate into plasma cells in vitro [25]. The culture conditions required for the production of autoantibodies from naïve B cells obtained from NMOSD patients included IL-2 as well as CD40L, TNF-α, IL-1β, IL-21, and a Toll-like receptor 7 agonist [11]. In accordance with these findings, we demonstrated CD25^+^ naïve B cells differentiated more efficiently than CD25^−^ naïve B cells when stimulated with BCR cross-linking, CD40L, IL-21, and IL-2. These results suggested that CD25^+^ naïve B cells are committed to differentiate into antibody-secreting cells, rather than a “naïve” population. Furthermore, their expansion may result in an increase of SMB cells and plasmablasts and the continuous production of autoantibodies in NMOSD patients. Autocrine IL-2 was reported to be involved in the survival and differentiation of T cells [32–34]. However, to the best of our knowledge, there is no evidence for the production of IL-2 from B cells. In addition, our RNA-seq analysis showed that B cells did not express IL-2 mRNA (data not shown). Therefore, we assumed that T cells were the major source of IL-2 in the differentiation of naïve B cells.

CD19^+^CD27^−^IgD^−^ DNB cells are another candidate antibody-secreting cell precursor. DNB cells were reported to be increased in the peripheral blood of SLE patients [35, 36]. Recently, autoreactive CD27^−^IgD^−^CXCR5^−^CD11c^+^ DN2 cells were reported to be expanded in SLE patients and differentiated into antibody-secreting cells under Toll-like receptor 7 signals [15]. DN2 cells are thought to be differentiated from CD27^−^IgD^+^CXCR5^−^CD11c^+^ activated naïve B cells, which are also increased in SLE patients. In our analysis, CD25^+^ naïve B cells did not express CD11c, suggesting they are a distinct population from the previously reported activated naïve B cells. In autoimmune diseases, multiple pathways might be involved in the differentiation of naïve B cells to antibody-secreting cells, and further studies are needed to elucidate which pathway may be dominant in each disease.

T_PH_ cells are a recently reported novel subpopulation of CD4^+^ helper T cells, which induce the differentiation of B cells into antibody-secreting cells [21] and were identified in the peripheral blood and synovial tissues of rheumatoid arthritis patients. T_PH_ cells produce IL-21 and activate B cells to produce antibodies, similar to T_FH_ cells. However, unlike T_FH_ cells, T_PH_ cells do not express CXCR5, which is required to enter germinal centers, but do express other chemokine receptors, CCR2, CXCR1, and CCR5, allowing migration into inflamed tissues [21]. These findings suggest that T_PH_ cells are involved in extrafollicular T–B cell interactions and B cell differentiation. Several studies recently demonstrated an increase of T_PH_ cells in the peripheral blood of patients with various autoimmune diseases [22–24, 37, 38]. In the current analysis, we report a significant increase of T_PH_ cells in the peripheral blood of NMOSD patients, which was not observed in MS and MOG-AD patients. Furthermore, T_PH_ cells from NMOSD patients induced the differentiation of SMB cells into antibody-secreting cells more efficiently than T_PH_ cells from HC, and this reaction was dependent on IL-21. These results indicated that T_PH_ cell-SMB cell interactions in extra-follicle regions, such as the intrathecal space might be important for the production of pathogenic AQP4-Ab. In addition to the abnormalities of B cells, numbers of abnormal T cells are assumed to be increased and to enhance the production of pathogenic antibodies in autoimmune diseases including NMOSD.

MOG is a glycoprotein exclusively expressed on the plasma membrane of oligodendrocytes and myelin in the CNS. Recent advances in detection methods using cell-based assays revealed that MOG-Ab was present in the peripheral blood of various inflammatory CNS diseases, especially pediatric diseases including acute disseminated encephalomyelitis [39]. However, the pathogenicity of MOG-Ab has not been fully demonstrated [40]. MOG-Ab positive NMOSD patients were reported to have different clinical features from AQP4-Ab positive NMOSD patients in terms of disease course, responsiveness to therapy including corticosteroids, and prognosis, suggesting that different pathomechanisms may exist between the two diseases [41]. Recently, it was reported that T_FH_ cells in the peripheral blood of MOG-Ab seropositive patients were significantly increased compared with HCs [42]. However, we did not observe a significant difference in the frequencies of T_PH_, T_FH_, and B cell subsets between MOG-AD patients and HCs in contrast to AQP4-Ab seropositive NMOSD patients. Our results suggest that the pathomechanisms of MOG-Ab production are different from those of AQP4-Ab positive NMOSD, in which the pathogenic autoantibody is continuously produced and other coexisting autoimmune diseases occur more frequently than MOG-AD [41, 43]. Because MOG was reported to not be expressed in the thymus [44], MOG-Ab may be produced secondarily during inflammation of the CNS. Further studies are needed to clarify the role of MOG-Ab in the pathogenesis of neuroinflammatory diseases.

## Conclusions

We revealed that B cell subsets in the peripheral blood and CSF of NMOSD patients were skewed into antibody-secreting cells and that SMB cells from NMOSD had an enhanced potential to differentiate into antibody-secreting cells. Therefore, the transcriptional profiles of B cell lineages were skewed towards an antibody-secreting cell-like phenotype. The transcriptional dysregulation of B cell lineages occurred in the early phase of maturation in NMOSD patients, and IL-2 induced genes were upregulated in naïve B cells. Furthermore, CD25^+^ naïve B cells were increased in the naïve B cells of NMOSD patients and had higher potential to differentiating into antibody-secreting cells compared with CD25^−^ naïve B cells, indicating CD25^+^ naïve B cells were committed to differentiate into antibody-secreting cells. These results shed light on a novel candidate precursor antibody-secreting cell in an antibody-related autoimmune disease and may represent a new therapeutic target.

## Supplementary Information

**Additional file 1: Figure S1.** Analysis of B cell subsets in PBMCs. (A) Gating strategy for B cell subsets. (B) Frequencies of B cell subsets among PBMCs (Kruskal-Wallis test). The box plot indicates the first and third quartiles and the middle line indicates the median. Whiskers indicate the minimum and maximum. *P < 0.05, **P < 0.01.

**Additional file 2: Figure S2.** Analysis of T cell subsets in PBMCs. (A) Gating strategy for T_PH_ cells and T_FH_ cells. (B) Frequencies of T cell subsets among PBMCs (Kruskal-Wallis test). The box plot indicates the first and third quartiles and the middle line indicates the median. Whiskers indicate the minimum and maximum. *P < 0.05, *** *P* < 0.005.

**Additional file 3: Figure S3.** Analysis of T cell subsets in PBMCs. Frequencies of CD25^+^ B cell subsets among PBMCs (Mann-Whitney *U*-test). The box plot indicates the first and third quartiles and the middle line indicates the median. Whiskers indicate the minimum and maximum. *P < 0.05.

**Additional file 4**: **Table S1 and Table S2**. Antibodies for flowcytometry and primer sequence for RT-qPCR analysis.

## Data Availability

The data sets used and analyzed during the current study are available from the corresponding author on reasonable request.

## Abbreviations

AQP4: Aquaporin 4
BCR: B cell receptor
CNS: Central nervous system
CSF: Cerebrospinal fluid
DEG: Differentially expressed gene
DNB: Double negative B
HC: Healthy control
MOG-AD: Myelin oligodendrocyte glycoprotein antibody-associated disorders
MS: Multiple sclerosis
NMOSD: Neuromyelitis optica spectrum disorder
PCA: Principal component analysis
PBMC: Peripheral blood mononuclear cells
SLE: Systemic lupus erythematosus
SMB: Switched memory B
