# Supplementary figures and images for: Dysregulated B cell differentiation towards antibody-secreting cells in neuromyelitis optica spectrum disorder
Source: J Neuroinflammation. 2022 Jan 6;19:6. doi: 10.1186/s12974-021-02375-w (PMC8740356; doi:10.1186/s12974-021-02375-w)

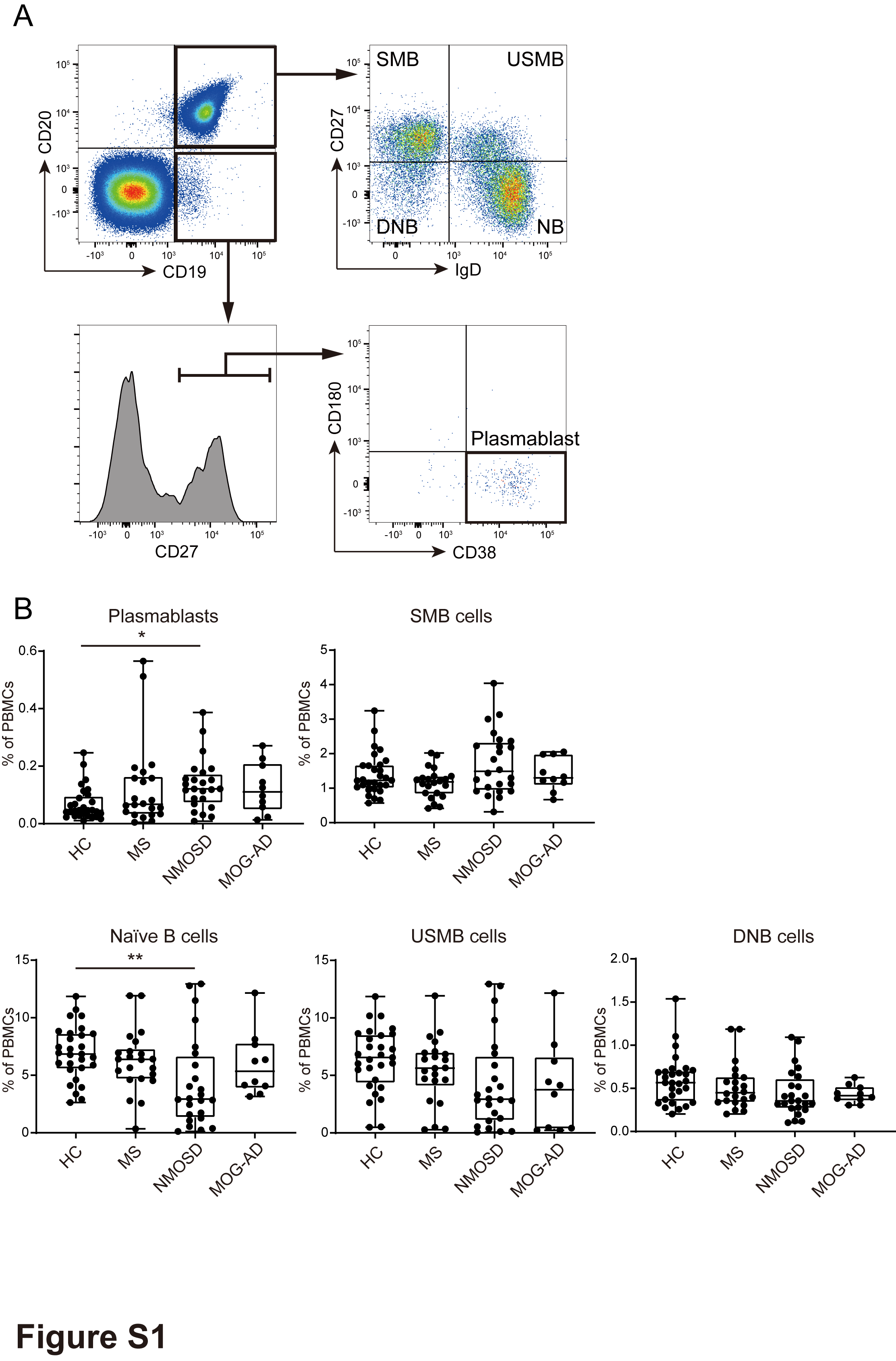

Supplement: Supplementary file 1 — Additional file 1: Figure S1. Analysis of B cell subsets in PBMCs. (A) Gating strategy for B cell subsets. (B) Frequencies of B cell subsets among PBMCs (Kruskal-Wallis test). The box plot indicates the first and third quartiles and the middle line indicates the median. Whiskers indicate the minimum and maximum. *P < 0.05, **P < 0.01. [file 12974_2021_2375_MOESM1_ESM.tif]

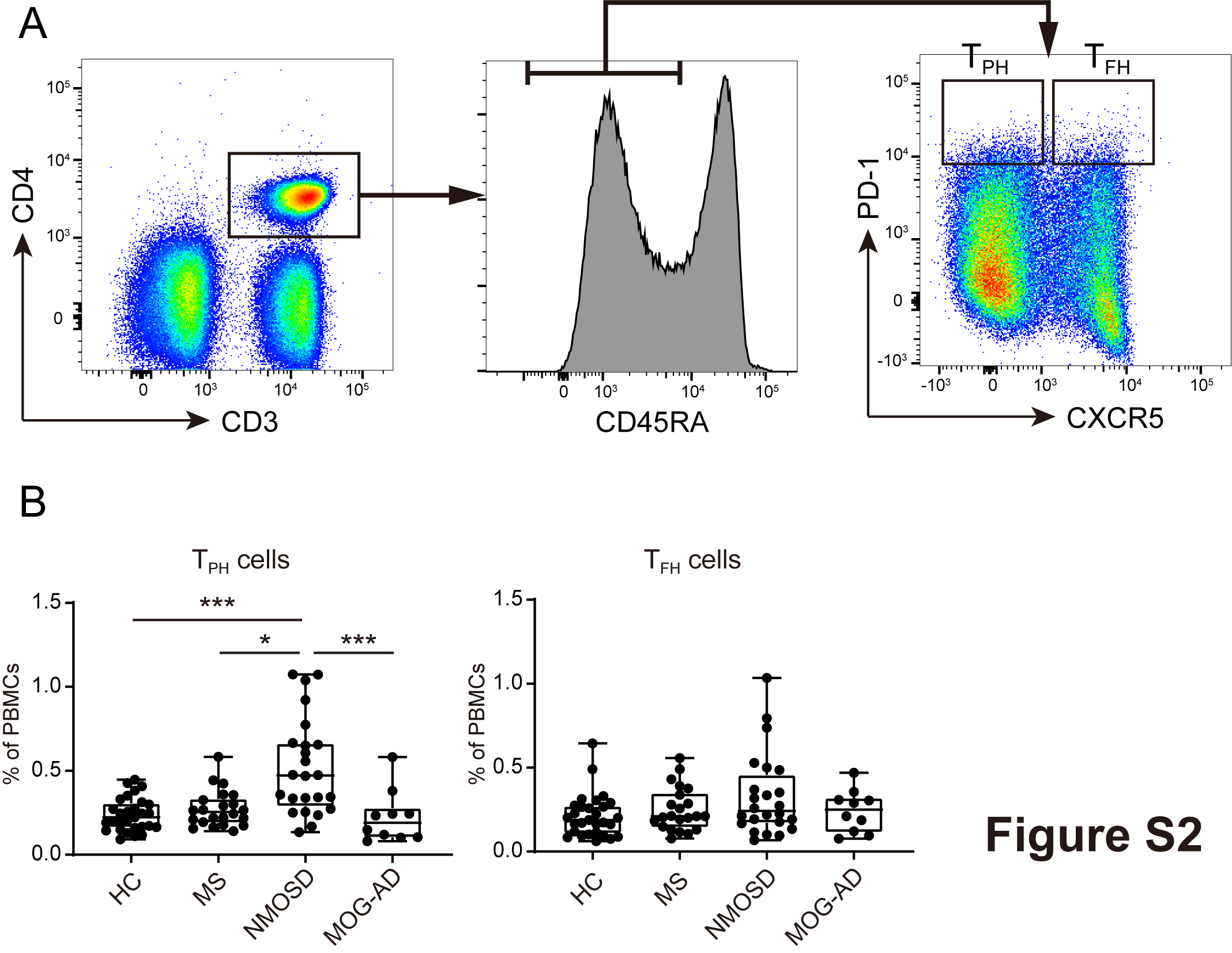

Supplement: Supplementary file 2 — Additional file 2: Figure S2. Analysis of T cell subsets in PBMCs. (A) Gating strategy for TPH cells and TFH cells. (B) Frequencies of T cell subsets among PBMCs (Kruskal-Wallis test). The box plot indicates the first and third quartiles and the middle line indicates the median. Whiskers indicate the minimum and maximum. *P < 0.05, *** P < 0.005. [file 12974_2021_2375_MOESM2_ESM.tif]

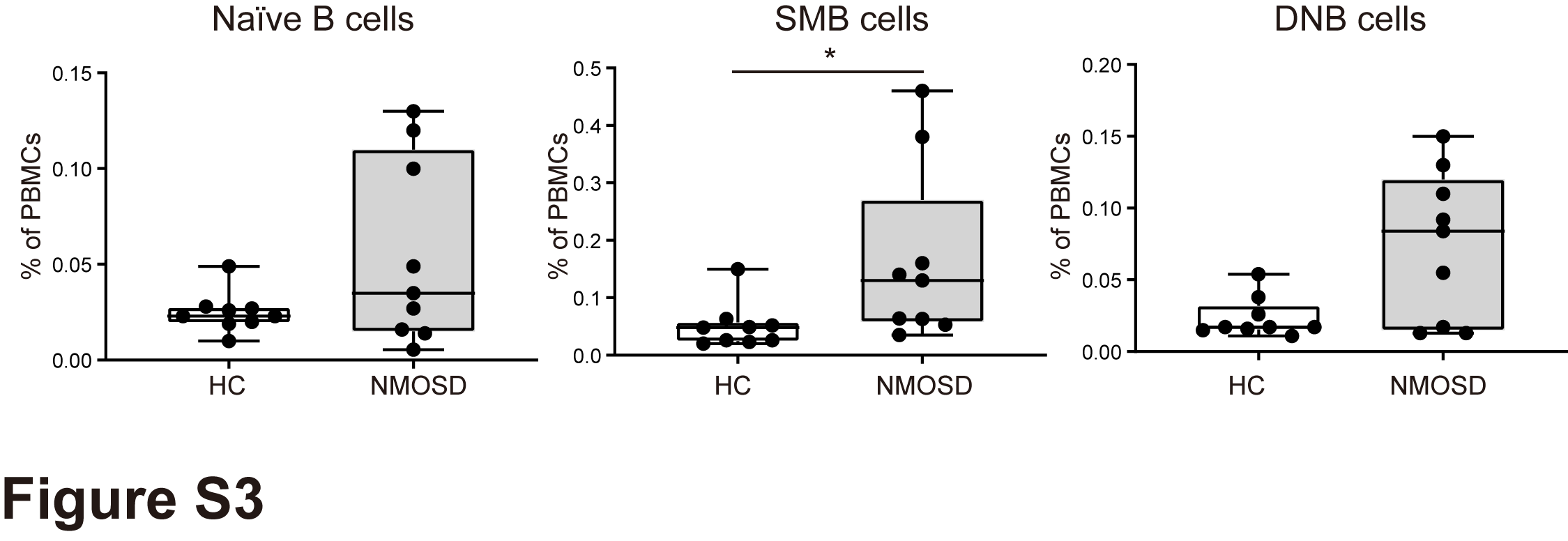

Supplement: Supplementary file 3 — Additional file 3: Figure S3. Analysis of T cell subsets in PBMCs. Frequencies of CD25+ B cell subsets among PBMCs (Mann-Whitney U-test). The box plot indicates the first and third quartiles and the middle line indicates the median. Whiskers indicate the minimum and maximum. *P < 0.05. [file 12974_2021_2375_MOESM3_ESM.tif]
