# Supplementary material for: Dysregulated B cell differentiation towards antibody-secreting cells in neuromyelitis optica spectrum disorder
Source: J Neuroinflammation. 2022 Jan 6;19:6. doi: 10.1186/s12974-021-02375-w (PMC8740356; doi:10.1186/s12974-021-02375-w)
Supplement: Supplementary file 4 — Additional file 4: Table S1 and Table S2. Antibodies for flowcytometry and primer sequence for RT-qPCR analysis. [file 12974_2021_2375_MOESM4_ESM.docx]

**Supplemental table 1. Antibodies for flowcytometry**

| Antigen | fluorochrome | Company |
| --- | --- | --- |
| B cell analysis | | |
| CD19 | PE-CF-594 | BioLegend |
| CD20 | Alexa700 | BioLegend |
| CD27 | APC-Fire750 | BioLegend |
| CD38 | FITC | Beckman Coulter, Indianapolis, IN |
| CD180 | PE | BioLegend |
| IgD | Brilliant Violet 421 | BioLegend |
| T cell analysis | | |
| CD3 | Brilliant Violet 421 | BioLegend |
| CD4 | APC-H7 | BD Pharmingen |
| CD45RA | Brilliant Violet 605 | BioLegend |
| CXCR5 | PE-CF-594 | BioLegend |
| PD-1 | PE | BioLegend |
| Cultured cell analysis | | |
| CD19 | PE-CF-594 | BioLegend |
| CD20 | Alexa700 | BioLegend |
| CD27 | APC-Fire750 | BioLegend |
| CD38 | FITC | Beckman Coulter |
| IgD | Brilliant Violet 421 | BioLegend |

**Supplemental table 2. Primer sequence for RT-qPCR analysis**

| gene | Forward primer (5’ → 3’) | Reverse primer (5’ → 3’) |
| --- | --- | --- |
| *ACTB* | CACTCTTCCAGCCTTCCTTCC | GCGTACAGGTCTTTGCGGATG |
| *BCL6* | TCCGTGCCCATGTGCTTATC | TGCAGGTTACACTTCTCACAATGG |
| *IL2RA* | TTATCAGTGCGTCCAGGGAT | CTCTTCACCTGGAAACTGACTGG |
| *PRDM1* | CATGACCGGCTACAAGACCC | TCAGGTGGACCTTCAGATTGG |
